# Axillary Management Trends and Survival in Men Undergoing Mastectomy with Positive Sentinel Nodes

**DOI:** 10.1245/s10434-025-18501-4

**Published:** 2025-10-13

**Authors:** Elizabeth M. Fish, Ian Whittall, Walker Lyons, Richard J. Bleicher, Rebecca M. Shulman, Cecilia Chang, Alycia L. So, Andrea S. Porpiglia, Allison A. Aggon, Austin D. Williams

**Affiliations:** 1 Division of Breast Surgery, Department of Surgical Oncology, Fox Chase Cancer Center, Philadelphia, PA USA; 2 Department of Surgery, Einstein Medical Center, Philadelphia, PA USA; 3 Department of Radiation Oncology, Fox Chase Cancer Center, Philadelphia, PA USA; 4 Research Institute, NorthShore University Health System, Evanston, IL USA

**Keywords:** Male breast cancer, Invasive breast cancer, Axillary management, Nodal positivity, AMAROS, Mastectomy, Post-mastectomy radiation, Axillary dissection

## Abstract

**Background:**

Men are often diagnosed with node-positive breast cancer and treated with mastectomy because of a lack of screening and an unfavorable tumor-to-breast ratio. The AMAROS trial showed no difference in outcomes between axillary lymph node dissection (ALND) and axillary radiation in women with cT1-2N0 breast cancer with positive sentinel lymph nodes (+SLNs). Axillary management in men remains unstandardized, so we assessed current trends and outcomes.

**Methods:**

Males with cT1-2N0M0 breast cancer undergoing mastectomy with one to two +SLNs were identified from the National Cancer Database (2018–2021). Patients were stratified by axillary management. Postmastectomy radiotherapy (PMRT) included chest wall and axillary fields. Management strategies and overall survival were analyzed.

**Results:**

Among 445 patients, 25% had no further axillary treatment, 22% underwent ALND, 29% PMRT, and 24% ALND+PMRT. Patients with two +SLNs more often underwent ALND+PMRT (43% vs. 19%, *p* < 0.001). The use of PMRT rose over time (23–36%), whereas ALND alone declined (27–12%). Additional positive nodes were found in 31% of ALND cases, with no difference between ALND and ALND+PMRT. Performance of ALND delayed PMRT (194 vs. 133 days from diagnosis, *p* < 0.001). On multivariable analysis, two +SLNs predicted ALND+PMRT (odds ratio 2.5, *p* = 0.006). Older age (*p* < 0.001) and two +SLNs (*p* = 0.03) were linked to worse overall survival, whereas axillary management was not (*p* = 0.23).

**Conclusion:**

Although axillary strategies are proven safe and effective in women, their extrapolation to men is inconsistent. Half of men undergoing mastectomy are undertreated or overtreated, underscoring the need for multidisciplinary consensus and prospective male-specific data to guide care and reduce morbidity.

**Supplementary Information:**

The online version contains supplementary material available at 10.1245/s10434-025-18501-4.

Male breast cancer (MBC) is a relatively rare and under-researched disease, accounting for less than 1% of breast cancers overall, with an estimated 2800 cases and 510 deaths in 2025.^1^ This rarity has prevented the development of randomized trials specifically evaluating the treatment of MBC, and few men have been included in broader breast cancer studies. Management guidelines for MBC have, therefore, largely been extrapolated from studies of women with breast cancer.^2^

Despite many similarities, there are notable differences in the presentation and tumor biology between male and female breast cancer. Given the lack of screening, MBC tends to be diagnosed at a later stage than breast cancer in women,^3^ resulting in a higher tumor-to-breast ratio in most cases and leading to recommendations for mastectomy rather than breast conservation in a higher proportion of patients, despite breast-conserving surgery (BCS) being shown as a safe option. ^4^ MBC is also more commonly hormone receptor-positive (HR+) and human epidermal growth factor receptor 2-negative (HER2−), potentially influencing disease progression, treatment decisions, and therapeutic response in ways distinct from in women.^5^ These clinical differences lead to a lack of standardization in the management of MBC despite treatment decisions being based on standardized algorithms.^4^

Axillary management in MBC has also deviated from modern protocols of de-escalation. Prior studies have demonstrated that men have been more commonly treated with axillary lymph node dissection (ALND) across all nodal stages,^6^ suggesting that there are opportunities for evidence-based de-escalation in MBC. Since 1988, breast cancer trials have aimed to de-escalate axillary surgery. The National Surgical Adjuvant Breast and Bowel Project (NSABP) B-04 study showed that omitting ALND in patients with cN0 disease did not impact survival.^7^ The American College of Surgeons Oncology Group (ACOSOG) Z0011 trial found that women with one to two positive sentinel lymph nodes (+SLNs) undergoing BCS plus radiation had similar overall survival (OS) and disease-free survival (DFS) whether or not they received ALND.^7,8^ Similarly, the landmark AMAROS (After Mapping of the Axilla, Radiotherapy or Surgery?) trial showed no difference in outcomes between ALND and axillary radiation in women with cT1-2N0 breast cancer and one or more +SLNs (95% of whom had either one or two +SLNs), with a concomitant reduction in morbidity when ALND was omitted.^9^ Notably, neither of these studies included men with breast cancer, and the integration of the results into the treatment of MBC has lagged compared with the treatment of women with breast cancer.

Mastectomy, the most common surgical approach for MBC, was performed in 17% of the AMAROS study population^9^ and expanded the opportunity to omit ALND beyond the BCS population studied in ACOSOG Z0011. However, the degree to which these data are currently being applied to the management of MBC and whether ALND omission leads to worse outcomes remains unknown. Therefore, our objective was to evaluate current trends and outcomes in the axillary management of men undergoing upfront mastectomy with limited nodal disease and to explore whether the findings of the AMAROS trial are being applied.

## Methods

After institutional review board approval, we performed a retrospective analysis of the National Cancer Database (NCDB). The NCDB is a joint collaboration between the American College of Surgeons and the American Cancer Society in which patient-level data are collected from all patients with cancer seen at Commission on Cancer-accredited programs,^10^ representing approximately 70% of cancer cases in the USA.^11^

From the NCDB breast participant user file, we identified male patients aged ≥ 18 years with cT1-2N0M0 invasive breast cancer, undergoing mastectomy with sentinel lymphadenectomy (SLNB) and had one to two +SLNs between 2018 and 2021 (Supplemental Fig. 1). We excluded patients who were pN0, underwent neoadjuvant systemic therapy or neoadjuvant radiation, had sarcoma or phyllodes tumors, or had a prior cancer diagnosis. Additional exclusions included patients with unknown axillary surgery, pathologic N stage, or adjuvant radiation status, and those who received chest wall radiation alone or had unknown regional nodal irradiation. Radiation data were extracted from the NCDB, with post-mastectomy radiation (PMRT) encompassing the chest wall and regional lymph nodes, including axillary, supraclavicular, and/or internal mammary sites.

We evaluated the clinicopathologic features of the cohort, stratifying by the number of +SLNs (one or two), and the type of axillary therapy beyond SLNB: none, completion ALND (cALND), PMRT alone, and cALND+PMRT. We then assessed the trends in axillary management over time from 2018 to 2021, as well as predictors of axillary treatment type with univariate and multivariable analyses. Predictors of axillary treatment type were assessed after excluding patients who had no further axillary treatment after SLNB. We then compared the use of adjuvant systemic therapy among men with HR+, HER2− breast cancer stratified by axillary therapy. Finally, we compared OS between the axillary management groups for the entire cohort and those with one or two +SLNs, stratified by type of axillary therapy, and created models to determine predictors of worse OS.

### Statistical Analysis

Comparisons between groups were made using the chi-squared test, Student’s t-test, and analysis of variance, as appropriate. Univariate and multivariable logistic regression models, adjusting for pertinent clinicopathologic and demographic features, were performed. OS was estimated using Kaplan–Meier curves and a Cox proportional hazards model. Statistical analysis was performed using SAS 9.4 (SAS Institute Inc., Cary, NC, USA). All tests were two-sided, and *p*-values < 0.05 were considered statistically significant.

## Results

### Study Cohort

We identified 712 men aged ≥ 18 years who underwent mastectomy with SLNB for cT1-2N0M0 invasive breast cancer between 2018 and 2021. After applying our exclusion criteria, 445 patients were included in the analysis, of whom 80% had one +SLN and 20% had two +SLNs (Table 1). The average number of SLNs examined was three. The SLN groups were very similar, with no differences in age, race, comorbidity index, clinical tumor stage, receptor subtype, or tumor histology. When compared with patients with one +SLN, more patients with two +SLNs were from neighborhoods with lower education attainment (44% vs. 50% of patients came from populations with ≤ 9.0% no high school degrees, *p* = 0.01), had high tumor grade (51% vs. 36%, *p* = 0.02), and a higher, though not significant, rate of lymphovascular invasion (56% vs. 49%, *p* = 0.14). Patients with two +SLNs underwent more axillary dissections overall than patients with one (57% vs. 42%, *p* < 0.001), and they were treated more often with cALND+PMRT (41% vs. 19%, *p* < 0.001).

**Table 1:** Clinicopathologic features of men with cT1-2N0 breast cancer undergoing upfront mastectomy with sentinel lymphadenectomy with one to two positive sentinel lymph nodes (SLNs) stratified by number of positive SLNs

Characteristics	Overall	Positive SLNs		*p*-value
		1	2	
n	445	354	91	
Age, years	65.4 ± 11.9	65.4 ± 12.1	65.4 ± 11.2	0.99
Race			0.41	
White	357 (80.2)	283 (79.9)	74 (81.3)	
Black	65 (14.6)	54 (15.3)	11 (12.1)	
Asian	–	–	–	
Other/unknown	–	–	–	
Charlson/Deyo Score				0.92
0	321 (72.1)	258 (72.9)	63 (69.2)	
1	76 (17.1)	59 (16.7)	17 (18.7)	
2	18 (4.0)	14 (4.0)	4 (4.4)	
≥ 3	30 (6.7)	23 (6.5)	7 (7.7)	
Insurance status			0.05	
Not insured	–	–	–	
Private insurance	190 (42.7)	149 (42.1)	41 (45.1)	
Medicaid	26 (5.8)	15 (4.2)	11 (12.1)	
Medicare	214 (48.1)	177 (50.0)	37 (40.7)	
Other government	–	–	–	
Unknown	–	–	–	
No high school degree				0.01
≥ 15.3%	–	49 (13.8)	–	
9.1–15.2%	88 (19.8)	61 (17.2)	27 (29.7)	
5.0–9.0%	111 (24.9)	97 (27.4)	14 (15.4)	
< 5.0%	105 (23.6)	79 (22.3)	26 (28.6)	
Unknown	–	68 (19.2)	–	
Institution type				0.53
Community cancer center	–	–	–	
Comprehensive community cancer program	169 (38.0)	133 (37.6)	36 (39.6)	
Academic/research program	141 (31.7)	117 (33.1)	24 (26.4)	
Integrated Network Cancer Program	88 (19.8)	66 (18.6)	22 (24.2)	
Unknown	–	–	–	
Clinical tumor stage				0.73
cT1	237 (53.3)	190 (53.7)	47 (51.6)	
cT2	208 (46.7)	164 (46.3)	44 (48.4)	
Receptor subtype			0.49	
HR+/HER2−	412 (92.6)	328 (92.7)	84 (92.3)	
HER2+	27 (6.1)	20 (5.6)	7 (7.7)	
TNBC	–	–	–	
Unknown	–	–	–	
Tumor histology				0.59
Ductal	440 (98.9)	349 (98.6)	91 (100.0)	
Lobular	–	–	–	
Unknown	–	–	–	
Tumor grade				0.02
Low	–	–	–	
Intermediate	242 (54.4)	199 (56.2)	43 (47.3)	
High	174 (39.1)	128 (36.2)	46 (50.5)	
Unknown	–	–	–	
Lymphovascular invasion				0.14
Absent	177 (39.8)	147 (41.5)	30 (33.0)	
Present	223 (50.1)	172 (48.6)	51 (56.0)	
Unknown	45 (10.1)	35 (9.9)	10 (11.0)	
Number of SLNs examined	3.1 ± 2.4	3.0 ± 2.5	3.2 ± 1.8	0.52
Axillary treatment after SLNB				< 0.001
None	113 (25.4)	103 (29.1)	10 (11.0)	
cALND alone	96 (21.6)	81 (22.9)	15 (16.5)	
PMRT alone	131 (29.4)	102 (28.8)	29 (31.9)	
cALND+PMRT	105 (23.6)	68 (19.2)	37 (40.7)	

Data are presented as mean ± standard deviation or n (%) unless otherwise indicated.

cALND, completion axillary lymph node dissection; HER2−, human epidermal growth factor receptor 2-negative; HR+ , hormone receptor positive; PMRT, post-mastectomy radiation; SLNB, sentinel lymphadenectomy; TNBC, triple-negative breast cancer.

### Axillary Surgery and Radiation

When stratifying the analytic cohort by axillary therapy approach, 25% underwent no additional treatment after SLNB, 21% underwent cALND alone, 29% underwent PMRT alone, and 24% had cALND+PMRT (Table 2). From 2018 to 2021, the use of cALND alone decreased from 27 to 12%, whereas the use of PMRT alone increased from 23 to 36% (Fig. 2), though the overall trend was not significant (*p* = 0.14). The mean age of the study population was 65 years; patients who underwent no additional treatment were older, and patients who underwent cALND+PMRT were younger (68 and 62.7 years, respectively, *p* = 0.007). Similar to the SLN group analysis, there were very few differences in clinicopathologic features between axillary therapy groups. However, there were notable differences in axillary therapy when SLN groups were compared: among patients with one +SLN, 29% underwent no further axillary treatment, 23% underwent cALND, 29% had PMRT alone, and 19% had cALND+PMRT (*p* < 0.001). Among patients with two +SLNs, only 11% underwent no further axillary treatment, 16% underwent cALND, 32% had PMRT alone, and 41% underwent cALND+PMRT (*p* < 0.001).

**Table 2:** Clinicopathologic features of men with cT1-2N0 breast cancer undergoing upfront mastectomy with sentinel lymphadenectomy with one to two positive sentinel nodes stratified by axillary therapy

Characteristic	Overall	None	cALND alone	PMRT alone	cALND+PMRT	*p*-value
	445	111	96	131	107	
Age, years	65.4 ± 11.9	68.3 ± 12.9	65.3 ± 11.9	65.1 ± 11.6	62.7 ± 11.5	0.007
Race						0.17
White	357 (80.2)	80 (72.1)	78 (81.3)	112 (85.5)	87 (81.3)	
Black	65 (14.6)	23 (20.7)	13 (13.5)	17 (13.0)	12 (11.2)	
Asian	–	–	–	–	–	
Other/unknown	–	–	–	–	–	
Charlson/Deyo Score						0.83
0	321 (72.1)	80 (72.1)	70 (72.9)	98 (74.8)	73 (68.2)	
1	76 (17.1)	19 (17.1)	12 (12.5)	22 (16.8)	23 (21.5)	
2	18 (4.0)	–	–	–	–	
≥ 3	30 (6.7)	–	–	–	–	
Insurance status						0.08
Not insured	–	–	–	–	–	
Private insurance	190 (42.7)	45 (40.5)	35 (36.5)	60 (45.8)	50 (46.7)	
Medicaid	26 (5.8)	1 (0.9)	7 (7.3)	8 (6.1)	10 (9.3)	
Medicare	214 (48.1)	60 (54.1)	52 (54.2)	58 (44.3)	44 (41.1)	
Other government	–	–	–	–	–	
Unknown	–	–	–	–	–	
No high school degree						0.01
≥ 15.3%	58 (13.0)	24 (21.6)	12 (12.5)	15 (11.5)	7 (6.5)	
9.1–15.2%	88 (19.8)	23 (20.7)	14 (14.6)	25 (19.1)	26 (24.3)	
5.0–9.0%	111 (24.9)	24 (21.6)	35 (36.5)	28 (21.4)	24 (22.4)	
< 5.0%	105 (23.6)	27 (24.3)	15 (15.6)	35 (26.7)	28 (26.2)	
Unknown	83 (18.7)	13 (11.7)	20 (20.8)	28 (21.4)	22 (20.6)	
Institution type						0.39
Community cancer center	38 (8.5)	–	–	–	–	
Comprehensive community cancer program	169 (38.0)	47 (42.3)	34 (35.4)	52 (39.7)	36 (33.6)	
Academic/research program	141 (31.7)	36 (32.4)	36 (37.5)	33 (25.2)	36 (33.6)	
Integrated Network Cancer Program	88 (19.8)	15 (13.5)	17 (17.7)	33 (25.2)	23 (21.5)	
Unknown	–	–	–	–	–	
Clinical tumor stage						0.39
cT1	237 (53.3)	62 (55.9)	57 (59.4)	65 (49.6)	53 (49.5)	
cT2	208 (46.7)	49 (44.1)	39 (40.6)	66 (50.4)	54 (50.5)	
Receptor subtype						0.46
HR+/HER2−	412 (92.6)	98 (88.3)	91 (94.8)	124 (94.7)	99 (92.5)	
HER2+	27 (6.1)	–	–	–	–	
TNBC	–	–	–	–	–	
Unknown	–	–	–	–	–	
Tumor histology						0.35
Ductal	440 (98.9)	109 (98.2)	96 (100.0)	129 (98.5)	106 (99.1)	
Lobular	–	–	–	–	–	
Unknown	–	–	–	–	–	
Tumor grade						0.17
Low	–	–	–	–	–	
Intermediate	242 (54.4)	54 (48.6)	51 (53.1)	81 (61.8)	56 (52.3)	
High	174 (39.1)	46 (41.4)	36 (37.5)	44 (33.6)	48 (44.9)	
Unknown	–	–	–	–	–	
Lymphovascular invasion						0.07
Absent	177 (39.8)	53 (47.7)	42 (43.8)	48 (36.6)	34 (31.8)	
Present	223 (50.1)	47 (42.3)	44 (45.8)	72 (55.0)	60 (56.1)	
Unknown	45 (10.1)	11 (9.9)	10 (10.4)	11 (8.4)	13 (12.1)	
Positive SLNs						< 0.001
1	354 (79.6)	103 (92.8)	81 (84.4)	102 (77.9)	68 (63.6)	
2	91 (20.4)	10 (9.0)	15 (15.6)	29 (22.1)	37 (34.6)	
Additional positive nodes*						< 0.001
Yes	62 (30.5)		17 (17.7)		45 (42.1)	
No	141 (69.5)		79 (82.3)		62 (57.9)	
Additional LNs examined*	10.4 ± 8.3		10.4 ± 8.8		10.4 ± 7.8	0.98
Additional LNs positive*	1.2 ± 3.3		0.6 ± 2.5		1.7 ± 3.8	
Total LNs examined*	13.6 ± 8.2		13.8 ± 8.9		13.4 ± 7.6	
Total LNs positive*	2.4 ± 3.3		1.7 ± 2.5		3.0 ± 3.8	

Data are presented as mean ± standard deviation or n (%) unless otherwise indicated.

cALND, completion axillary lymph node dissection; HER2−, human epidermal growth factor receptor 2-negative; HR+ , hormone receptor positive; LN, lymph node; PMRT, post-mastectomy radiation; SLN, sentinel lymph node; TNBC, triple-negative breast cancer.

*Results suppressed: NCDB does not permit aggregate results for cell sizes < 10.

Overall, 31% of patients who had cALND had additional positive nodes, of whom the significant majority (73%) subsequently received PMRT rather than having cALND alone (*p* < 0.001). Overall, a mean number of 10 additional LNs were removed at the time of cALND, with a mean 1.2 additional +LNs found.

Performance of cALND was associated with a mean delay to initiating radiotherapy of 60 days (cALND+PMRT: 194 days from diagnosis vs. PMRT alone: 133 days, *p* < 0.001). Among the patients who underwent PMRT, either alone or with cALND, there was no significant difference in radiation (XRT) volume, XRT to draining LNs, XRT treatment modality, planning technique, dose per fraction, number of fractions, total dose, XRT treatment days, or reasons for XRT ending early (Supplemental Table 1).

We created univariate and multivariable logistic regression models to assess factors associated with each type of axillary therapy. On multivariable analysis, there were only two independent predictors of undergoing ALND: having two +SLNs (vs. one +SLN, odds ratio [OR] 2.43; 95% confidence interval [CI] 1.18–4.99, *p* = 0.02, Table 3) and the omission of PMRT (vs. inclusion, OR > 1000; 95% CI 183 to > 1000, *p* < 0.001, Table 3). Similarly, the two independent predictors for the use of PMRT were having two +SLNs (vs. one +SLN, OR 2.81; 95% CI 1.20–6.59, *p* = 0.02, Table 4) and the omission of ALND (vs. inclusion, OR 200; 95% CI 14to > 1000, *p* < 0.001, Table 4). Lastly having two +SLNs was the only independent predictor of undergoing cALND+PMRT on univariate and multivariable analysis (OR 2.47; 95% CI 1.29–4.70, *p* = 0.006, Supplemental Table 2). Thus, age, race, education, clinical tumor stage, receptor subtype, tumor grade, and lymphovascular invasion were not predictors of axillary surgery or radiation in this cohort.

**Table 3:** Factors predictive of axillary lymph node dissection (ALND) among men with cT1-2N0 breast cancer undergoing upfront mastectomy with one to two positive sentinel nodes (eliminated patients who had no additional axillary therapy)

Characteristic	Univariate			Multivariable		
	OR	95% CI	*p*-value	OR	95% CI	*p*-value
Age, years	1.01	0.99–1.03	0.36	0.99	0.96–1.02	0.44
Race/ethnicity						
White (ref)	–	–	–	–	–	–
Black	0.99	0.51–1.92	0.98	1.04	0.36–2.95	0.95
Asian	3.85	0.61–24.35	0.15	12.78	0.36–460.39	0.16
No high school degree						
≥ 15.3% (ref)	–	–	–	–	–	–
9.1–15.2%	1.26	0.54–2.93	0.59	2.46	0.74–8.18	0.14
5.0–9.0%	1.66	0.74–3.74	0.22	2.44	0.73–8.10	0.15
< 5.0%	0.97	0.43–2.19	0.95	2.49	0.75–8.29	0.14
Clinical tumor stage						
cT1 (ref)	–	–	–	–	–	–
cT2	0.83	0.54–1.29	0.42	1.22	0.63–2.37	0.56
Receptor subtype						
HR+/HER2− (ref)	–	–	–	–	–	–
HER2 +	1.61	0.57–4.56	0.37	0.96	0.23–3.94	0.95
Tumor grade						
Low (ref)	–	–	–	–	–	–
Intermediate	0.69	0.25–1.89	0.47	0.44	0.09–2.21	0.32
High	0.99	0.35–2.79	0.98	0.83	0.16–4.34	0.82
Lymphovascular invasion						
Absent (ref)	–	–	–	–	–	–
Present	0.91	0.57–1.46	0.71	1.09	0.55–2.18	0.80
Positive SLNs						
1 (ref)	–	–	–	–	–	–
2	1.27	0.76–2.12	0.37	2.43	1.18–4.99	0.02
PMRT						
No (ref)	–	–	–	–	–	–
Yes	0.004	< 0.001–0.07	< 0.001	< 0.001	< 0.001–0.06	< 0.001

Data are presented as mean ± standard deviation or n (%) unless otherwise indicated.

CI, confidence interval; HER2−/+, human epidermal growth factor receptor 2-negative/positive; HR+, hormone receptor positive; OR, odds ratio; PMRT, post-mastectomy radiation; SLN, sentinel lymph node.

**Table 4:** Factors predictive of post-mastectomy radiation (PMRT) use among men with cT1-2N0 breast cancer undergoing upfront mastectomy with one to two positive sentinel lymph nodes (eliminated patients who had no additional axillary therapy)

Characteristic	Univariate			Multivariable		
	OR	95% CI	*p*-value	OR	95% CI	*p*-value
Age, years	1.01	0.99–1.03	0.37	0.99	0.96–1.02	0.41
Race/ethnicity						
White (ref)	–	–	–	–	–	–
Black	0.86	0.43–1.74	0.67	0.73	0.25–2.19	0.58
Asian	0.48	0.13–1.84	0.28	1.09	0.14–8.36	0.93
No high school degree						
≥ 15.3% (ref)	–	–	–	–	–	–
9.1–15.2%	1.97	0.79–4.93	0.15	2.14	0.59–7.72	0.25
5.0–9.0%	0.82	0.36–1.87	0.64	1.08	0.32–3.66	0.90
< 5.0%	2.28	0.93–5.58	0.07	2.54	0.69–9.32	0.16
Clinical tumor stage						
cT1 (ref)	–	–	–	–	–	–
cT2	1.48	0.92–2.39	0.11	1.28	0.59–2.77	0.54
Receptor subtype						
HR+/HER2− (ref)	–	–	–	–	–	–
HER2+	1.01	0.35–2.87	0.99	1.12	0.19–6.47	0.90
Tumor grade						
Low (ref)	–	–	–	–	–	–
Intermediate	2.67	1.00–7.10	0.05	1.35	0.30–6.04	0.70
High	2.53	0.93–6.89	0.07	1.87	0.41–8.61	0.42
Lymphovascular Invasion						
Absent (ref)	–	–	–	–	–	–
Present	1.53	0.93–2.54	0.10	1.42	0.69–2.94	0.35
Positive SLNs						
1 (ref)	–	–	–	–	–	–
2	2.11	1.14–3.91	0.02	2.81	1.20–6.59	0.02
ALND						
No (ref)	–	–	–	–	–	–
Yes	0.004	< 0.001–0.07	< 0.001	0.005	< 0.001–0.07	< 0.001

Data are presented as mean ± standard deviation or n (%) unless otherwise indicated.

ALND, axillary lymph node dissection; CI, confidence interval; HER2−, human epidermal growth factor receptor 2-negative; HR+ , hormone receptor positive; OR, odds ratio; SLN, sentinel lymph node.

### Adjuvant Systemic Therapy

We then assessed the use of adjuvant systemic therapy after limiting the cohort to those with HR+ /HER2− breast cancer given the small proportion of patients (6%) who had other subtypes. Overall, the 21-gene recurrence score (RS) assay was performed in 48% of patients with a mean score of 17 (Table 5). The RS was performed most commonly for patients who had PMRT alone and least commonly for patients having cALND+PMRT (46% vs. 25% of all patients with an RS, *p* = 0.045), though there was no difference in mean RS between the axillary therapy groups (*p* = 0.35). The use of adjuvant chemotherapy was most common among patients who had cALND+PMRT (71% vs. none: 16%, cALND alone: 27%, PMRT alone: 37%, *p* < 0.001, Table 5). Most patients (84%) had adjuvant endocrine therapy, but patients who underwent no further axillary treatment were least likely to receive this (71% vs. cALND alone: 77%, PMRT alone: 93%, cALND+PMRT: 91%, *p* < 0.001). Taken together, patients receiving adjuvant PMRT with or without ALND were more likely to undergo adjuvant systemic therapy than those who did not have PMRT.

**Table 5:** Use of adjuvant systemic therapy among men with hormone receptor-positive (HR+)/human epidermal growth factor receptor 2-negative (HER2−) cT1-2N0 breast cancer undergoing upfront mastectomy with sentinel lymphadenectomy with one or two positive sentinel nodes stratified by axillary therapy

Characteristics	Overall	None	cALND alone	PMRT alone	cALND+PMRT	*p*-value
	412	98	91	124	99	
Recurrence score						
Not performed	213 (51.7)	50 (51.0)	47 (51.7)	54 (43.6)	62 (62.6)	0.045
Performed	199 (48.3)	48 (49.0)	44 (48.4)	70 (56.5)	37 (37.4)	
Mean ± SD	16.9 ± 10.5	14.6 ± 9.3	18.5 ± 13.2	17.1 ± 10.5	17.5 ± 8.5	0.35
Chemotherapy						< 0.001
No/unknown	250 (60.7)	82 (83.7)	66 (72.5)	78 (62.9)	29 (29.3)	
Yes	157 (38.1)	16 (16.3)	25 (27.5)	46 (37.1)	70 (70.7)	
Endocrine therapy						< 0.001
No	47 (11.4)	–	–	–	–	
Yes	345 (83.7)	70 (71.4)	70 (76.9)	115 (92.7)	90 (90.9)	
Unknown	20 (4.9)	–	–	–	–	

cALND, completion axillary lymph node dissection; PMRT, post-mastectomy radiation; SD, standard deviation.

### Survival Results and Predictors

**Fig. 1:**
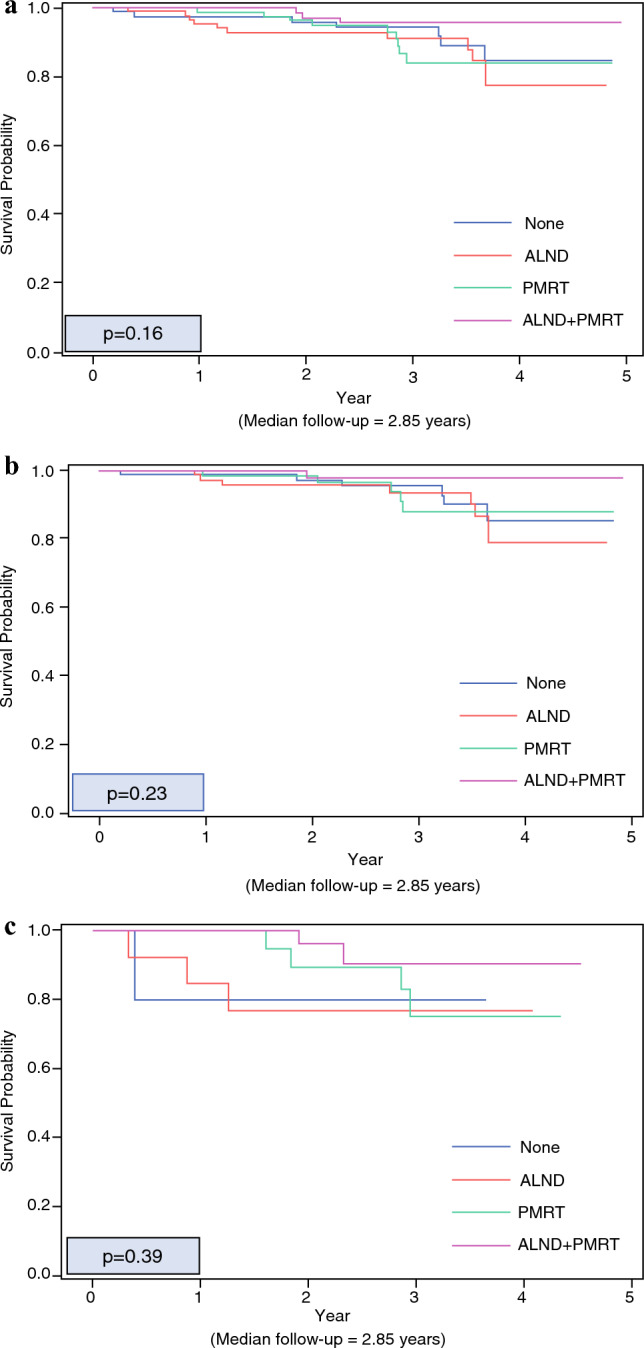
**a** Kaplan Meier curve of OS for whole cohort stratified by axillary therapy. **b** Kaplan Meier curve of OS for 1 +SLN stratified by axillary therapy. **c** Kaplan Meier curve of OS for 2 +SLN stratified by axillary therapy

With a median follow-up of 2.9 years, OS was ≥80% in all groups. There was no difference in unadjusted OS between the axillary treatment groups in the overall cohort (Fig. 1a, *p* = 0.16), and in subgroups stratified by one +SLN (Fig. 1b *p* = 0.23) and two +SLN (Fig. 1c, *p* = 0.39). On univariate analysis, the only significant predictors of worse OS were for older age (hazard ratio 1.07; 95% CI 1.03–1.11, *p* < 0.001) and having two +SLNs (vs. one +SLN, hazard ratio 2.30; 95% CI 1.08–4.91, *p* = 0.03, Table 6). We could not perform multivariable analysis because of the low event rate (number of deaths =  30).

**Table 6:** Factors predictive of overall survival (OS) among men with cT1-2N0 breast cancer undergoing upfront mastectomy with one to two positive sentinel nodes (univariate analysis)

Factor	Hazard ratio	95% CI	*p*-value
Age (years)	1.07	1.03–1.11	< 0.001
Race/ethnicity			
White (ref)	–	–	–
Black	1.45	0.59–3.56	0.41
Asian	1.77	0.32–9.64	0.51
Charlson/Deyo Score			
0 (ref)	–	–	–
1	2.11	0.91–4.93	0.08
2	1.22	0.22–6.85	0.82
≥ 3	2.77	0.95–8.09	0.06
No high school degree			
≥ 15.3% (ref)	–	–	–
9.1–15.2%	1.11	0.33–3.74	0.87
5.0–9.0%	1.41	0.45–4.46	0.56
< 5.0%	0.44	0.10–1.87	0.26
Clinical tumor stage			
cT1 (ref)	–	–	–
cT2	1.82	0.88–3.78	0.11
Receptor subtype			
HR+/HER2− (ref)	–	–	–
HER2+	1.35	0.36–5.04	0.65
Tumor grade			
Low (ref)	–	–	–
Intermediate	0.51	0.15–1.69	0.27
High	0.66	0.20–2.22	0.50
Lymphovascular invasion			
Absent (ref)	–	–	–
Present	1.48	0.70–3.13	0.31
Positive SLNs			
1 (ref)	–	–	–
2	2.30	1.08–4.91	0.03
Axillary treatment after SLNB			
None (ref)	–	–	–
cALND alone	1.42	0.55–3.68	0.47
PMRT alone	1.27	0.47–3.43	0.63
cALND+PMRT	0.41	0.11–1.53	0.18

cALND, completion axillary lymph node dissection; CI, confidence interval; HER2−, human epidermal growth factor receptor 2 negative; HR+ , hormone receptor positive; PMRT, post-mastectomy radiation; SLN, sentinel lymph node.

## Discussion

In this retrospective analysis of a national database, over half of men with one to two +SLNs undergoing mastectomy currently experience axillary overtreatment or undertreatment based on the AMAROS paradigm, which established the noninferiority of PMRT alone compared with ALND. Specifically, 24% of patients in this cohort received ALND+PMRT, whereas 25% had no additional axillary treatment after SLNB. Patients with two +SLNs were more likely to undergo ALND+PMRT and axillary dissection overall, with the number of +SLNs emerging as the sole independent predictor of receiving ALND+PMRT. Although recurrence scores were similar across treatment groups, adjuvant chemotherapy was more frequently used in those receiving ALND+PMRT. Importantly, OS did not differ by axillary treatment strategy, even when stratified by SLN burden. These findings highlight a pressing need for standardized axillary management and provider education in MBC.

De-escalation of axillary management in patients with limited nodal disease has been driven by the substantial morbidity associated with both ALND and PMRT. ALND carries high rates of complications, including paresthesia (53%), lymphedema (25%), seroma formation (16%), limited shoulder mobility (24%), and chronic pain (28%).^12,13^ PMRT, which includes chest wall and regional nodal irradiation, is also associated with lymphedema (7%), brachial plexopathy (0.4–9%), and pneumonitis (5%).^14–17^ When both modalities are combined, these risks are compounded. One prospective study reported a 7% higher 2-year cumulative incidence of lymphedema with combined ALND+PMRT compared with ALND alone, and other studies cite even higher rates.^17,18^ Functional impairment has similarly been shown to exceed 80% with combined treatment, representing a 3- to 4-fold increase over ALND alone.^19^ These findings have led to efforts aimed at tailoring axillary treatment to reduce overtreatment in appropriately selected patients. However, data guiding such decisions remain limited in MBC.

The AMAROS trial investigated whether axillary radiotherapy (RT) could serve as a less morbid alternative to ALND in patients with early breast cancer and one or more +SLNs. The trial demonstrated no significant difference in 5-year axillary recurrence rates between the ALND and RT groups (0.54% vs. 1.03%, respectively), confirming comparable regional control. OS and DFS were also similar between the two arms. However, morbidity differed notably: at 5 years, lymphedema occurred in 23% of patients who underwent ALND compared with only 11% in the RT group. Patients receiving RT also experienced better shoulder mobility and less arm swelling, highlighting the quality-of-life benefits of radiation. These findings supported axillary RT as a safe and less invasive alternative to ALND for selected patients.^9^

We found some evidence of axillary de-escalation and the application of the AMAROS paradigm to the treatment of MBC in our study. Although not statistically significant, ALND+PMRT decreased over the study period and, when analyzed separately, the use of ALND decreased, whereas the use of PMRT alone increased. This is a trend also reported by Wang et al.^20^ in their NCDB analysis of women with one to two +LNs treated with mastectomy. They found that the use of ALND dropped from 47 to 18% between 2012 and 2021 and that PMRT alone rose from 10% to 37%. Notably, 21% of patients still received both ALND and PMRT. It is unclear whether the +LNs identified in this study were SLNs, since this variable was only distinct beginning in 2018. Regardless, given the well-documented morbidity associated with ALND, particularly when combined with radiation, this continued use of combination therapy, and ALND instead of PMRT warrants re-evaluation.

Although the use of genomic assays remains controversial in pN +  MBC, we found that nearly half of patients in our study had a 21-gene RS performed. Despite ALND+PMRT patients undergoing RS testing less frequently, RSs did not differ between axillary treatment groups. In our study, patients with HR+ /HER2− tumors who underwent ALND+PMRT were also more likely to receive adjuvant chemotherapy, which notably did not result in improved survival outcomes. Given the similar RSs between treatment groups, it may be inferred that the increased use of chemotherapy was not entirely driven by higher genomic risk. This pattern raises the possibility that clinicians may be influenced by the aggressiveness of local treatment, such as ALND+PMRT, when making decisions about systemic therapy, potentially leading to continued overtreatment. Given that all patients in our cohort were male, it is also possible that heightened concern about recurrence in this population has contributed to more aggressive adjuvant treatment strategies.

Recent trials have examined whether additional axillary treatment beyond SLNB is necessary for patients with one to two +SLNs. The SENOMAC^21^ and SINODAR-ONE multicenter, randomized trials evaluated the safety of omitting ALND in early-stage breast cancer. In SENOMAC, 5-year OS was 93% with SLNB alone versus 92% with ALND; DFS was similarly close (90% vs. 89%). SINODAR-ONE reported a 5-year OS of 99% in the SLNB group and 98% in the ALND group, with no significant DFS difference.^21,22^ Although both trials included male patients, their numbers were too small for meaningful subgroup analysis. Mastectomy rates were 36% in SENOMAC^21^ and 23% in SINODAR-ONE, and nearly 88% of SINODAR-ONE participants received adjuvant regional nodal radiation.^22^ Despite this, outcomes were comparable without additional axillary surgical intervention. Given these findings, and the lack of observed survival difference in our own male cohort between SLNB alone and ALND+PMRT, we must ask: should *any* further axillary treatment be pursued in males with one to two +SLNs? Since 25% of patients in our cohort had no further axillary treatment, it seems that many care teams believe this to be a safe approach and are ready for continued de-escalation for their male patients.

Axillary management in MBC remains unstandardized, largely because of the absence of male-specific data and guidelines. Current practices are extrapolated from female-focused trials, leading to significant variability in provider decisions. Inclusion of male patients in de-escalation trials and survey-based research on practice patterns will further inform best practices. Future work should focus on developing clinical guidelines tailored to male patients, supported by data from national or international registries. Provider education through CME programs and decision-support tools could also help bridge knowledge gaps. Although standardization is important, it must be balanced with flexibility for individualized decision-making. Multidisciplinary discussions remain crucial to ensure patient-centered care that considers both oncologic safety and patient preferences.

This study has several strengths and limitations. Utilizing the NCDB, which is a large, nationally representative dataset, we were able to examine contemporary trends in axillary management for MBC and evaluate their impact. However, given that MBC comprises < 1% of all breast cancer cases, our cohort size (N = 445) remains modest, though consistent with prior research. Our analysis was restricted to 2018–2021 data, as SLN details were only captured in the NCDB from 2018. Therefore, median follow-up was 2.9 years; as most tumors were HR+, late recurrences may not yet be evident, limiting our interpretation of survival outcomes. Furthermore, the database lacks granular data (such as the reason RS testing was pursued; the rate was somewhat higher than expected) and information on recurrence and treatment-related morbidity, restricting our ability to assess locoregional outcomes or complications by axillary approach or number of positive SLNs.

## Conclusion

Despite the availability of safe and effective management strategies for limited nodal disease in women, half of men with cT1-2N0M0 invasive breast cancer with one to two +SLNs undergoing mastectomy are being undertreated or overtreated according to the AMAROS paradigm. Although having two +SLNs is associated with worse OS, the choice of additional axillary therapy does not appear to affect survival. Multidisciplinary collaboration is essential to optimize outcomes and minimize morbidity for individual patients, and including men in future de-escalation trials is essential to provide data to standardize the approach to axillary therapy for men.

## Supplementary Information

Below is the link to the electronic supplementary material.

Supplementary file1 (DOCX 156 KB)
