# Supplementary material for: Axillary Management Trends and Survival in Men Undergoing Mastectomy with Positive Sentinel Nodes
Source: Ann Surg Oncol. 2025 Oct 13;33(2):1150–61. doi: 10.1245/s10434-025-18501-4 (PMC12765751; doi:10.1245/s10434-025-18501-4)
Supplement: Supplementary file 1 — Supplementary file1 (DOCX 156 KB) [file 10434_2025_18501_MOESM1_ESM.docx]

Supplemental Figure 1: STROBE Diagram


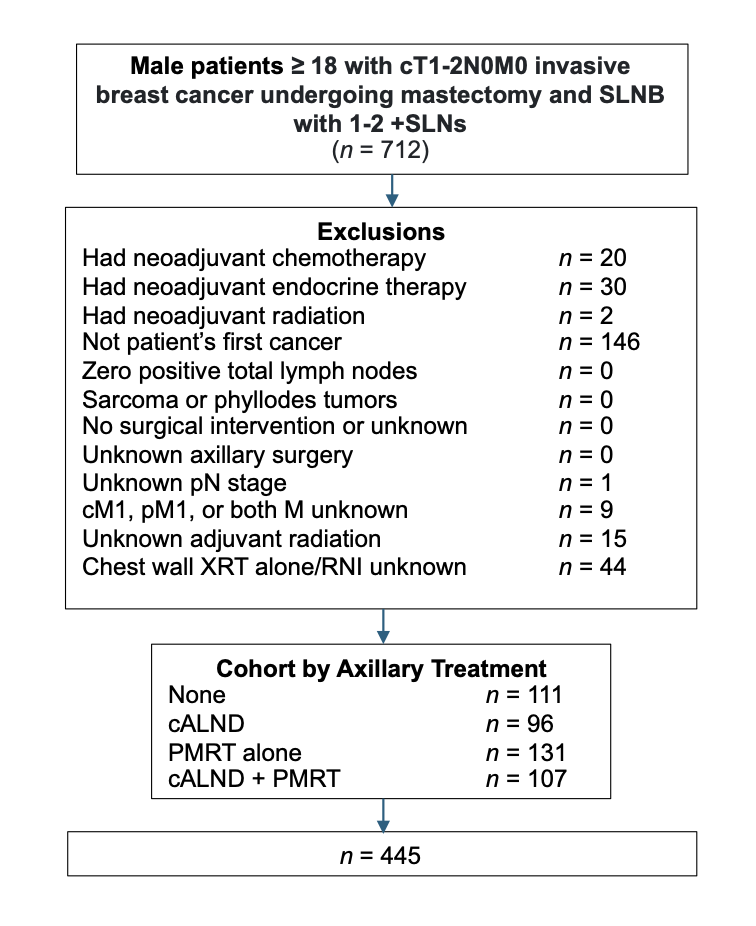


Supplemental Table 1: Radiation Variables from Table 1

|  |  | **Overall** | **PMRT alone** | **cALND + PMRT** | **p** |
| --- | --- | --- | --- | --- | --- |
| **n** |  | 238 | 131 | 107 |  |
| **XRT Total Dose**** | |  |  |  | 0.75 |
|  | <45 Gy | 10 (7.6) | 10 (7.6) | 9 (8.4) |  |
|  | ≥45 Gy | 119 (90.8) | 119 (90.8) | 92 (86.0) |  |
|  | Unknown | 2 (1.5) | 2 (1.5) | 6 (5.6) |  |
| **Radiation, days from Dx**, Mean ± SD** | | 160.6 ± 73.1 | 133.3 ± 60.8 | 194.0 ± 73.4 | <0.001 |
| **Radiation primary treatment volume**** | |  |  |  | 0.27 |
|  | Breast/Chest wall lymph node regions | 14 (5.9) | 10 (7.6) | 4 (3.7) |  |
|  | Breast (whole) | 27 (11.3) | 11 (8.4) | 16 (15.0) |  |
|  | Breast (partial) | 4 (1.7) | 2 (1.5) | 2 (1.9) |  |
|  | Chest wall | 192 (80.7) | 108 (82.4) | 84 (78.5) |  |
|  | Unknown | 1 (0.4) | 0 (0.0) | 1 (0.9) |  |
| **Radiation to draining lymph nodes**** | |  |  |  | 0.30 |
|  | None | 21 (8.8) | 15 (11.5) | 6 (5.6) |  |
|  | Neck lymph node regions | 6 (2.5) | 4 (3.1) | 2 (1.9) |  |
|  | Thoracic lymph node regions | 1 (0.4) | 1 (0.8) | 0 (0.0) |  |
|  | Neck and thoracic lymph node regions | 1 (0.4) | 1 (0.8) | 0 (0.0) |  |
|  | Breast/Chest wall lymph node regions | 192 (80.7) | 98 (74.8) | 94 (87.9) |  |
|  | Lymph node region, NOS | 2 (0.8) | 1 (0.8) | 1 (0.9) |  |
|  | Not applicable; Radiation primary treatment is lymph nodes | 15 (6.3) | 11 (8.4) | 4 (3.7) |  |
| **Radiation treatment modality**** | |  |  |  | 0.69 |
|  | External beam, NOS | 24 (10.1) | 11 (8.4) | 13 (12.1) |  |
|  | Photons | 209 (87.8) | 118 (90.1) | 91 (85.0) |  |
|  | Protons | 3 (1.3) | 1 (0.8) | 2 (1.9) |  |
|  | Electrons | 2 (0.8) | 1 (0.8) | 1 (0.9) |  |
| **Radiation external beam planning technique**** | |  |  |  | 0.23 |
|  | External beam, NOS | 52 (21.8) | 29 (22.1) | 23 (21.5) |  |
|  | Low energy x-ray/photon therapy | 2 (0.8) | 1 (0.8) | 1 (0.9) |  |
|  | 2-D therapy | 3 (1.3) | 0 (0.0) | 3 (2.8) |  |
|  | Conformal or 3-D conformal therapy | 158 (66.4) | 91 (69.5) | 67 (62.6) |  |
|  | Intensity modulated therapy | 23 (9.7) | 10 (7.6) | 13 (12.1) |  |
| **Radiation dose per fraction (Gy)**, Mean ± SD** | | 2.0 ± 0.4 | 2.0 ± 0.5 | 2.0 ± 0.4 | 0.60 |
| **Radiation number of fractions**, Mean ± SD** | | 26.7 ± 23.3 | 25.9 ± 21.8 | 27.6 ± 25.1 | 0.59 |
| **Radiation total dose (Gy)**, Mean ± SD** | | 47.9 ± 5.5 | 47.9 ± 5.2 | 47.8 ± 5.9 | 0.92 |
| **Radiation ended (days from start)**, Mean ± SD** | | 39.6 ± 9.6 | 39.3 ± 9.4 | 39.9 ± 10.0 | 0.64 |
| **Reason for radiation ending early**** | |  |  |  | 0.70 |
|  | Completed as prescribed | 122 (93.1) | 122 (93.1) | 101 (94.4) |  |
|  | Toxicity | 3 (2.3) | 3 (2.3) | 1 (0.9) |  |
|  | Contraindication | 1 (0.8) | 1 (0.8) | 0 (0.0) |  |
|  | Patient decision | 2 (1.5) | 2 (1.5) | 1 (0.9) |  |
|  | Reason not documented | 2 (1.5) | 2 (1.5) | 0 (0.0) |  |
|  | Unknown | 1 (0.8) | 1 (0.8) | 4 (3.7) |  |

Supplemental Table 2: Factors predictive of ALND+PMRT use among men with cT1-2N0 breast cancer undergoing upfront mastectomy with 1-2 positive sentinel nodes (eliminated patients who had no additional axillary therapy)

|  |  | **UNIVARIATE** | | | **MULTIVARIABLE** | | |
| --- | --- | --- | --- | --- | --- | --- | --- |
|  |  | ***OR*** | ***95% CI*** | ***p*** | ***OR*** | ***95% CI*** | ***p*** |
| **Age (years)** | | 1.02 | 1.00-1.04 | 0.07 | 0.99 | 0.96-1.01 | 0.31 |
| **Race/Ethnicity** | |  |  |  |  |  |  |
|  | White (ref) | - | - | - | - | - | - |
|  | Black | 0.89 | 0.44-1.82 | 0.75 | 0.93 | 0.38-2.29 | 0.87 |
|  | Asian | 1.78 | 0.47-6.79 | 0.4 | 2.85 | 0.41-20.09 | 0.29 |
| **No High School Degree** | |  |  |  |  |  |  |
|  | >=15.3% (ref) | - | - | - | - | - | - |
|  | 9.1% - 15.2% | 2.46 | 0.94-6.41 | 0.07 | 2.05 | 0.70-5.98 | 0.19 |
|  | 5.0% - 9.0% | 1.42 | 0.55-3.64 | 0.47 | 1.59 | 0.56-4.58 | 0.39 |
|  | < 5.0% | 2.07 | 0.81-5.30 | 0.13 | 2.34 | 0.81-6.76 | 0.12 |
| **Clinical Tumor Stage** | |  |  |  |  |  |  |
|  | cT1 (ref) | - | - | - | - | - | - |
|  | cT2 | 1.18 | 0.75-1.87 | 0.47 | 1.24 | 0.68-2.27 | 0.49 |
| **Receptor Subtype** | |  |  |  |  |  |  |
|  | HR+/HER2- (ref) | - | - | - | - | - | - |
|  | HER2+ | 1.75 | 0.67-4.58 | 0.25 | 1.06 | 0.30-3.80 | 0.93 |
| **Tumor Grade** | |  |  |  |  |  |  |
|  | Low (ref) | - | - | - | - | - | - |
|  | Intermediate | 1.89 | 0.55-6.47 | 0.31 | 0.78 | 0.20-3.00 | 0.72 |
|  | High | 2.67 | 0.77-9.26 | 0.12 | 1.43 | 0.37-5.52 | 0.6 |
| **Lymphovascular Invasion** | |  |  |  |  |  |  |
|  | Absent (ref) | - | - | - | - | - | - |
|  | Present | 1.36 | 0.83-2.25 | 0.23 | 1.28 | 0.70-2.35 | 0.43 |
| **Positive SLNs** | |  |  |  |  |  |  |
|  | 1(ref) | - | - | - | - | - | - |
|  | 2 | 2.38 | 1.42-3.97 | <0.001 | 2.47 | 1.29-4.70 | 0.006 |
